# Application of a Bioactive/Bioresorbable Three-Dimensional Porous Uncalcined and Unsintered Hydroxyapatite/Poly-d/l-lactide Composite with Human Mesenchymal Stem Cells for Bone Regeneration in Maxillofacial Surgery: A Pilot Animal Study

**DOI:** 10.3390/ma12050705

**Published:** 2019-02-27

**Authors:** Jingjing Sha, Takahiro Kanno, Kenichi Miyamoto, Yunpeng Bai, Katsumi Hideshima, Yumi Matsuzaki

**Affiliations:** 1 Department of Oral and Maxillofacial Surgery, Shimane University Faculty of Medicine, 89-1 Enya-Cho, Izumo, Shimane 693-8501, Japan; jsswjbnjw@gmail.com (J.S.); xyywq@126.com (Y.B.); hideg@med.shimane-u.ac.jp (K.H.); 2 Department of Cancer Biology, Shimane University Faculty of Medicine, 89-1 Enya-Cho, Izumo, Shimane 693-8501, Japan; miyaken@med.shimane-u.ac.jp (K.M.); matsuzak@med.shimane-u.ac.jp (Y.M.)

**Keywords:** maxillofacial defect, three-dimensional porous uncalcined and unsintered hydroxyapatite/poly-d/l-lactide composite scaffold, human mesenchymal stem cells, bone regeneration, blood supply, osteoconductivity

## Abstract

A novel three-dimensional (3D) porous uncalcined and unsintered hydroxyapatite/poly-d/l-lactide (3D-HA/PDLLA) composite demonstrated superior biocompatibility, osteoconductivity, biodegradability, and plasticity, thereby enabling complex maxillofacial defect reconstruction. Mesenchymal stem cells (MSCs)—a type of adult stem cell—have a multipotent ability to differentiate into chondrocytes, adipocytes, and osteocytes. In a previous study, we found that CD90 (Thy-1, cluster of differentiation 90) and CD271 (low-affinity nerve growth factor receptor) double-positive cell populations from human bone marrow had high proliferative ability and differentiation capacity in vitro. In the present study, we investigated the utility of bone regeneration therapy using implantation of 3D-HA/PDLLA loaded with human MSCs (hMSCs) in mandibular critical defect rats. Microcomputed tomography (Micro-CT) indicated that implantation of a 3D-HA/PDLLA-hMSC composite scaffold improved the ability to achieve bone regeneration compared with 3D-HA/PDLLA alone. Compared to the sufficient blood supply in the mandibular defection superior side, a lack of blood supply in the inferior side caused delayed healing. The use of Villanueva Goldner staining (VG staining) revealed the gradual progression of the nucleated cells and new bone from the scaffold border into the central pores, indicating that 3D-HA/PDLLA loaded with hMSCs had good osteoconductivity and an adequate blood supply. These results further demonstrated that the 3D-HA/PDLLA-hMSC composite scaffold was an effective bone regenerative method for maxillofacial boney defect reconstruction.

## 1. Introduction

Many patients suffer from mandibular defects due to benign or malignant tumors, trauma, and dysplastic pathologies [1], which can damage chewing function, mental health, and facial esthetics in patients. Therefore, the ability to perform high quality three-dimensional (3D) mandibular reconstruction is of critical importance [2]. Felice et al. [3] followed-up bone grafts in 115 patients with posterior mandibular atrophy for 2–7 years. The outcomes of those with heterologous and autogenous bone blocks were similar; thus, heterologous blocks may be preferable because no invasive harvesting is required. Tissue engineering represents a promising approach to solve the problems associated with tissue reconstruction [4]. Many synthetic scaffold composites afford significant clinical improvements, including the MaioRegen™ device placed after regenerative surgery to treat chondral and osteochondral lesions [5,6]. In daily clinical applications, ideal bone grafts should be off-the-shelf products that are easily handled, applied in a single step, and inexpensive. An ideal scaffold mimics bone morphology and the functional properties of original bony tissue, is biodegradable and biocompatible, supports early bone regeneration, and is gradually replaced by regenerating tissue [6]. Absorbable biodegradable polymers vary in terms of their physical and mechanical properties; these can be that engineered to optimize bone regeneration [7]. For example, synthetic β-tricalcium phosphate (β-TCP) has been used to reconstruct bone defects for several years, but is difficult to trim and does not wholly replace natural bone [8]. Therefore, β-TCP can only play an ideal role during oromandibular reconstruction.

A novel 3D porous uncalcined and unsintered hydroxyapatite/poly-d/l-lactide (3D-HA/PDLLA) material has been reported to have superior plasticity, biocompatibility, and osteoconductivity, as well as good biodegradability, both in vitro and in vivo. 3D-HA/PDLLA has been successfully implanted into animal femora and tibia [8,9,10]. Moreover, ectopic osteogenesis was observed in the implantation of 3D-HA/PDLLA loaded with bone marrow cells into muscles [11]. Therefore, it could potentially be developed for clinical applications as a novel bioresorbable scaffold in maxillofacial surgery.

Porous 3D-HA/PDLLA loaded with bone marrow aspirate (BMA) may be a useful alternative bone graft [12]. The high fusion ratio achieved with BMA may be attributed to a large number of cells, particularly mesenchymal stem cells (MSCs), which can be loaded into the porous 3D-HA/PDLLA [13]. Hernigou et al. found that bone healing depended on the numbers and proportions of MSCs and osteoblasts [14]. However, BMA cannot achieve adequate purity, and large quantities of MSCs cannot be obtained; therefore, we assessed the use of a high concentration of highly purified human MSCs (hMSCs) to replace BMA.

hMSCs are found in various fetal and adult human tissues, including the liver, term placenta, umbilical cord blood, and BMA [15], and can differentiate into myocytes, chondrocytes, adipocytes, and osteoblasts [16,17,18]. They are used to treat patients with bone and joint diseases including acute osteochondral fractures, spinal disk injuries, and rheumatoid arthritis, and inherited conditions [19]. Moreover, compared to embryonic stem cells, MSCs are not associated with a risk of malignant disease and no ethical problems arise [20]. MSCs are multipotent and minimally immunogenic; therefore, they are potential candidates for a variety of clinical applications [21,22]. Previously, we reported that double-positive cells of CD90 (Thy-1, cluster of differentiation 90) and CD271 (low-affinity nerve growth factor receptor) from human bone marrow mononuclear cells were highly contained colony-forming unit fibroblast cells, which had the trilineage potential to differentiate into chondrogenic, osteogenic, and adipogenic cells [15]. Our moderately expanding MSC clones (MECs) proliferating from single CD90/CD271 double-positive cells were highly purified and superselective compared with conventionally isolated hMSCs.

In the present study, we assessed the reconstruction of the critical mandibular defect in rats using 3D-HA/PDLLA-hMSC-derived regenerative biomaterial to further enhance and improve the bone-regeneration capacity of 3D-HA/PDLLA. 

## 2. Materials and Methods

### 2.1. The 3D-HA/PDLLA Composite Scaffold

**Figure 1:**
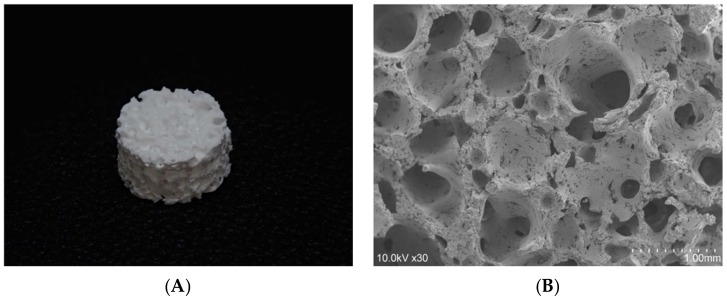
A photograph (**A**) and scanning electron micrograph (SEM) (**B**) of the three-dimensional (3D) porous uncalcined and unsintered hydroxyapatite/poly-d/l-lactide (3D-HA/PDLLA) composite scaffold.

The 3D-HA/PDLLA composite scaffold (Teijin Medical Technologies, Osaka, Japan) was composed of 30 wt.% poly-d/l-lactide (PDLLA) and 70 wt.% uncalcined and unsintered hydroxyapatite (u-HA) (viscosity-average molecular weight (Mv): 77 kDa; dextrorotatory-lactide acid/levorotatory-lactide acid (d/l) ratio: 50/50 mol%) matrix prepared via hot compression molding of nonwoven composite fibers. The calcium/phosphorus (Ca/P) molar ratio was 1.69, similar to that of pure hydroxyapatite (1.67). The 3D-HA/PDLLA composite scaffold had pores with 40–480 μm (average: 170 μm) pore size and 4.1 ± 0.4 MPa compressive strength (Figure 1B). The apparent densities of the pores in the scaffold were 70% [23]. In the present study, the 3D-HA/PDLLA composite scaffold was used as round patches with 4 mm in diameter and 2 mm in thickness (Figure 1A).

### 2.2. Preparation of Human Bone Marrow MSCs

Isolation of human bone marrow MSCs was described previously [15]. MECs were cultured on tissue culture plates containing low-glucose Dulbecco’s Modified Eagle’s Medium (Wako Pure Chemical Corp., Osaka, Japan) supplemented with 20% fetal bovine serum (Hyclone, South Logan, UT, USA), 10 mM HEPES (Nacalai, Kyoto, Japan), 1% penicillin/streptomycin (Wako Pure Chemical Corp., Osaka, Japan), and 20 ng/mL basic fibroblast growth factor. Cultured MECs were trypsinized and suspended with Hank’s Balanced Salt Solution (HBSS) supplemented with 2% fetal bovine serum.

### 2.3. The Critical Mandibular Defect Rat Model

**Figure 2:**
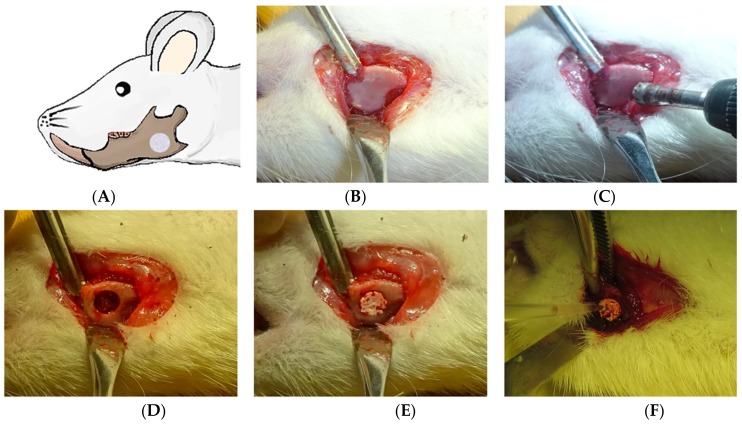
Images showing mandibular defect creation in vivo. (**A**) The mandibular defect model. (**B**) Incision and exposure of the mandible. (**C**) Drilling to create a defect in the middle part of the mandible (between the mandibular foramen and the margin). (**D**) The nonself-repairable critical defect of diameter 4 mm. (**E**) Implantation of 3D-HA/PDLLA patches into the defects of the Hank’s Balanced Salt Solution (HBSS) group and the two hMSCs groups. (**F**) Slow injection of HBSS or hMSCs onto the surface of the material in the HBSS group, the 1 × 10^4^ hMSCs group, and the 1 × 10^5^ hMSCs group, respectively.

Twenty-four male Sprague Dawley (SD) rats (average weight: 300–350 g; 10 weeks of age) were purchased from Charles River (Tokyo, Japan). To develop the mandibular defect rat model (Figure 2A), SD rats were anesthetized with pentobarbital (50 mg/kg) by intraperitoneal injection. An approximately 1-cm-long longitudinal incision was made in the mandible through the full thickness of the skin under standard aseptic conditions, and the muscles were separated with blunt forceps until the mandible surface was exposed (Figure 2B). On the mandibular body, we created a critical nonself-repairing defect 4 mm in diameter using a trephine bar (Figure 2C,D) [24]. The 24 SD rats were divided into four groups of 6 rats each: a no-transplantation group, a 3D-HA/PDLLA + HBSS group (HBSS group), a 3D-HA/PDLLA + 1 × 10^4^ hMSCs group (1 × 10^4^ hMSCs group), and a 3D-HA/PDLLA + 1 × 10^5^ hMSCs group (1 × 10^5^ hMSCs group). Briefly, 3D-HA/PDLLA composites were implanted to fill the defect in the HBSS group, 1 × 10^4^ hMSCs group, and 1 × 10^5^ hMSCs group (Figure 2E). Then, 10 µL volumes containing the HBSS, 1 × 10^4^ hMSCs, or 1 × 10^5^ hMSCs were injected using a P20 pipette tip according to the group sequence listed (Figure 2F). The no-transplantation group involved the defect model only. The wounds were closed by careful stitching of the skin layers. The rats awoke 1–2 h after the operation, and usually behaved and exhibited a normal appetite. Daily injections of tacrolimus (1 mg/kg/day) and ampicillin (20 mg/kg/day) were given to all rats until 2 or 4 weeks after the implantation to avoid immunological rejection and postoperative infections.

All animal experiments carefully followed the Guidelines for Care and Use of Laboratory Animals of the Shimane University Faculty of Medicine, and the protocol was approved by our institutional animal ethics committee (approval nos. IZ 27–126, IZ 30–68).

### 2.4. Microcomputed Tomography (Micro-CT) Analysis

Micro-CT (Siemens Inveon, Munich, Germany) mandibular radiographs were conducted, and all calculations were performed using ImageJ software (Media Cybernetics, Rockville, MD, USA). The density of 3D-HA/PDLLA is similar to that of cortical bone, whereas the density of new osteoid tissues (those formed within 4 weeks) is lower than that of 3D-HA/PDLLA. Using different threshold values, the material and newly formed osteoid tissue could be separated in each image. By adjusting the threshold and using the function of freehand selection and freehand line drawing options of ImageJ software, we measured the average fusion indices and the average areas of newly formed osteoid tissue in each Micro-CT layer. All images were independently evaluated by two blinded investigators to ensure data reliability.

The average fusion rate represented the average percentage of the material surface in contact with the host bone in each radiograph and was calculated as:

$$\mathrm{The}\;\mathrm{average}\;\mathrm{fusion}\;\mathrm{rate}\;=\;\frac{length\;of\;new\;bone\;fusion\;in\;the\;material\;surface\;}{total\;length\;of\;the\;material\;surface\;}\times 100\%.$$

The average fusion depth was the average depth of new osteoid tissue infiltrated into the material in each image. Average fusion rate and average fusion depth were used to evaluate the degree of fusion of the material and host bone. The average area of newly formed osteoid tissue was the quantity of newly formed osteoid tissue in each radiograph, which was used to assess the progress of ectopic bone formation.

**Figure 3:**
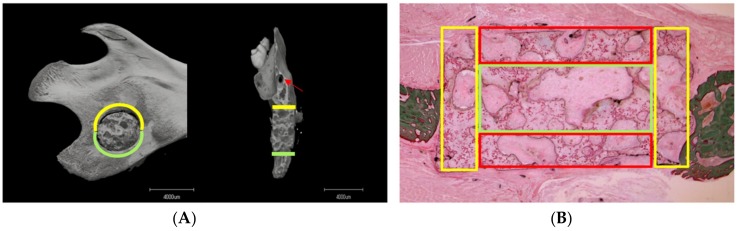
(**A**) The patterns of the superior and inferior regions of the critical mandibular defect. The yellow line defines the superior side (near the mandibular foramen), the green line the inferior side (near the mandibular bone margin as evident in the sagittal and coronal planes), and the red arrow shows the mandibular foramen. (**B**) The different pore areas. The pores in the yellow, red, and green grid squares represent connective pores (i.e., those located in the junction between the material and host bone), surrounding pores (i.e., those located at the buccal and lingual sides of the material), and central pores (i.e., those located in the center of the material), respectively.

The average fusion rate and average fusion depth between the mandibular critical defect on the superior and inferior sides were analyzed separately (Figure 3A), to determine whether blood supply and nutrition influenced ectopic bone regeneration.

### 2.5. Histological Analysis

Defective mandibular portions were excised from euthanized rats at 2 and 4 weeks. The samples were fixed with 10% neutral buffered formalin solution and dehydrated in a series of ethanol solutions (30, 50, 70, 80, 90, and 100% v/v) for 2 days. Then portions of the mandible were embedded in polyester resin (LR White Embedding Resin; London Resin, London, UK) and sectioned using a band saw (BS-300CP; EXAKT Apparatebau GmbH, Norderstedt, Germany) parallel to the coronal plane of the rat mandible. The surface of the slice was polished with diamond paper (MG-4000; EXAKT Apparatebau). For Villanueva Goldner staining (VG staining), specimens were stained with iron hematoxylin for 20 min, treated with 1% hydrochloric acid–ethanol solution, stained with Ponceau Fuchsin for 120 min, treated with 1% acetic acid, and stained with phosphotungstic acid–phosphomolybdic acid solution. They were then stained with Naphthol Green solution for 30 min and treated with 70–99.5% ethanol [25]. The samples were observed under a light microscope to evaluate bone regeneration. Nucleated cells (i.e., hMSCs, osteoblasts, osteoclasts, osteocytes, chondrocytes, macrophages, and so on) were counted (using ImageJ software) in three different areas of the material pores (i.e., the connective, surrounding, and central regions) (Figure 3B). For each specimen, all pore areas were examined under a light microscope (×40) by selecting six random fields. The area of each field was calculated using the Freehand Selection tool, and the Cell Counter tool was employed to count nucleated cell numbers. We then calculated the average number of nucleated cells per square millimeter. All slides were independently reviewed by two blinded investigators, and the average values taken as the nucleated cell counts.

### 2.6. Statistical Analysis

The Kruskal–Wallis H test was used to analyze the average fusion rate and average fusion depth. To explore differences in osteogenesis of the critical mandibular defects between the superior and inferior sides; we employed the Wilcoxon signed-rank test. To compare the quantity of newly formed osteoid tissue on Micro-CT and the nucleated cells count on VG staining, one-way analysis of variance and the LSD-*t* test were used. All statistical analyses were performed using SPSS statistical software (SPSS Japan Inc., Tokyo, Japan). All differences were considered significant at *p* < 0.05.

## 3. Results

### 3.1. Micro-CT Analysis

#### 3.1.1. Image Description

**Figure 4:**
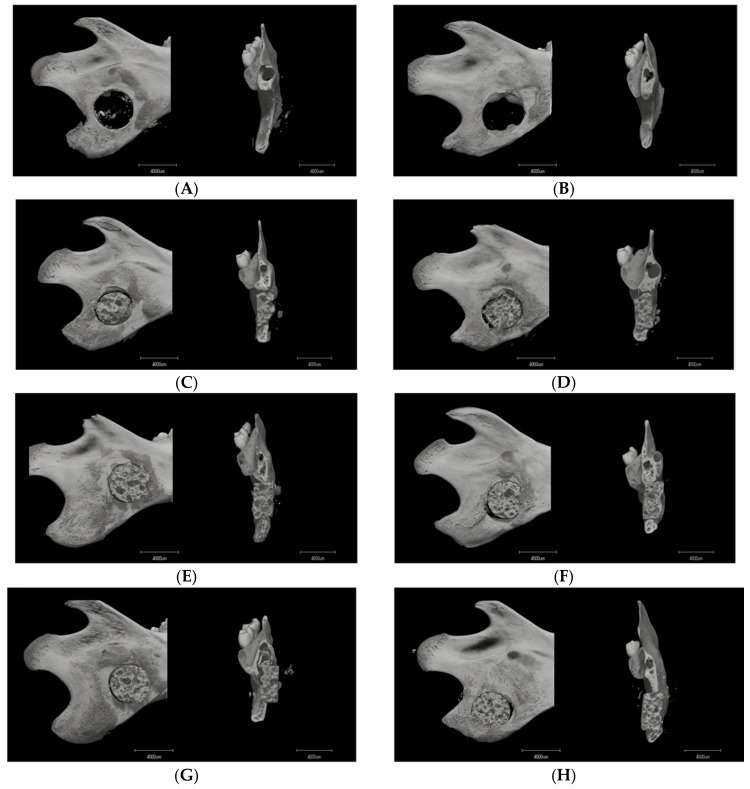
Microcomputed tomography images of the mandibular defects (sagittal and coronal images) at ((**A**,**C**,**E**,**G**); n = 3) 2 weeks and ((**B**,**D**,**F**,**H**); n = 3) 4 weeks. (**A**,**B**) Group 1: the no-transplantation group. (**C**,**D**) Group 2: the 3D-HA/PDLLA + HBSS group (HBSS group). (**E**,**F**) Group 3: the 3D-HA/PDLLA + 1 × 10^4^ hMSCs group (1 × 10^4^ hMSCs group). (**G**,**H**) Group 4: the 3D-HA/PDLLA + 1 × 10^5^ hMSCs group (1 × 10^5^ hMSCs group). Scale bar: 4000 µm.

Micro-CT imaging was performed at two and four weeks after surgery to analyze bone formation in the mandibular defect rats. No obvious bone formation was observed in rats in the no-transplantation group (Figure 4A,B), whereas the mandibular bone of the HBSS group was mildly fused (Figure 4C,D). In contrast, the implantation of the composite with hMSCs was more abundantly fused with the mandibular bone (Figure 4E–H). The fusion appeared at two weeks in the 1 × 10^4^ hMSCs group (Figure 4E), and was broader and denser at four weeks (Figure 4F). With the addition of 1 × 10^5^ hMSCs, the compact fusion was observed at two weeks (Figure 4G). At four weeks, the host bone closely fused with the composite, and the new bone surrounding the buccal–lingual side was shown using Micro-CT (Figure 4H).

#### 3.1.2. Material–Host Bone Combinations and the Quantity of Newly Formed Osteoid Tissue

**Figure 5:**
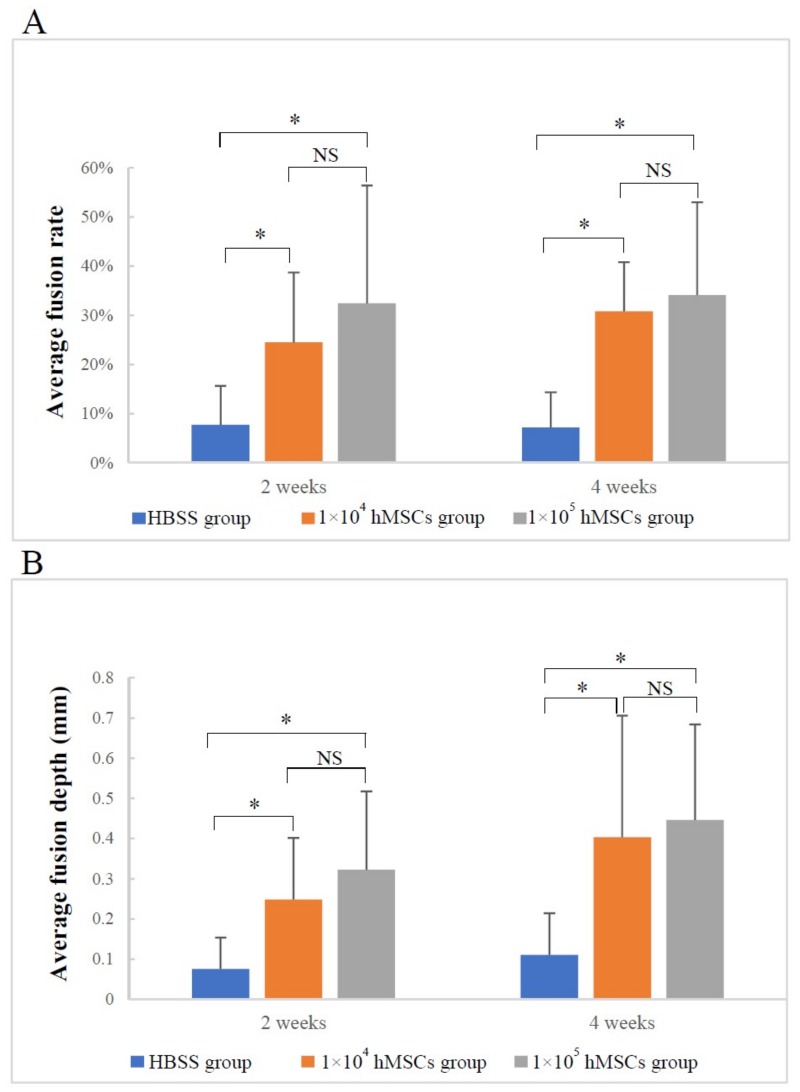

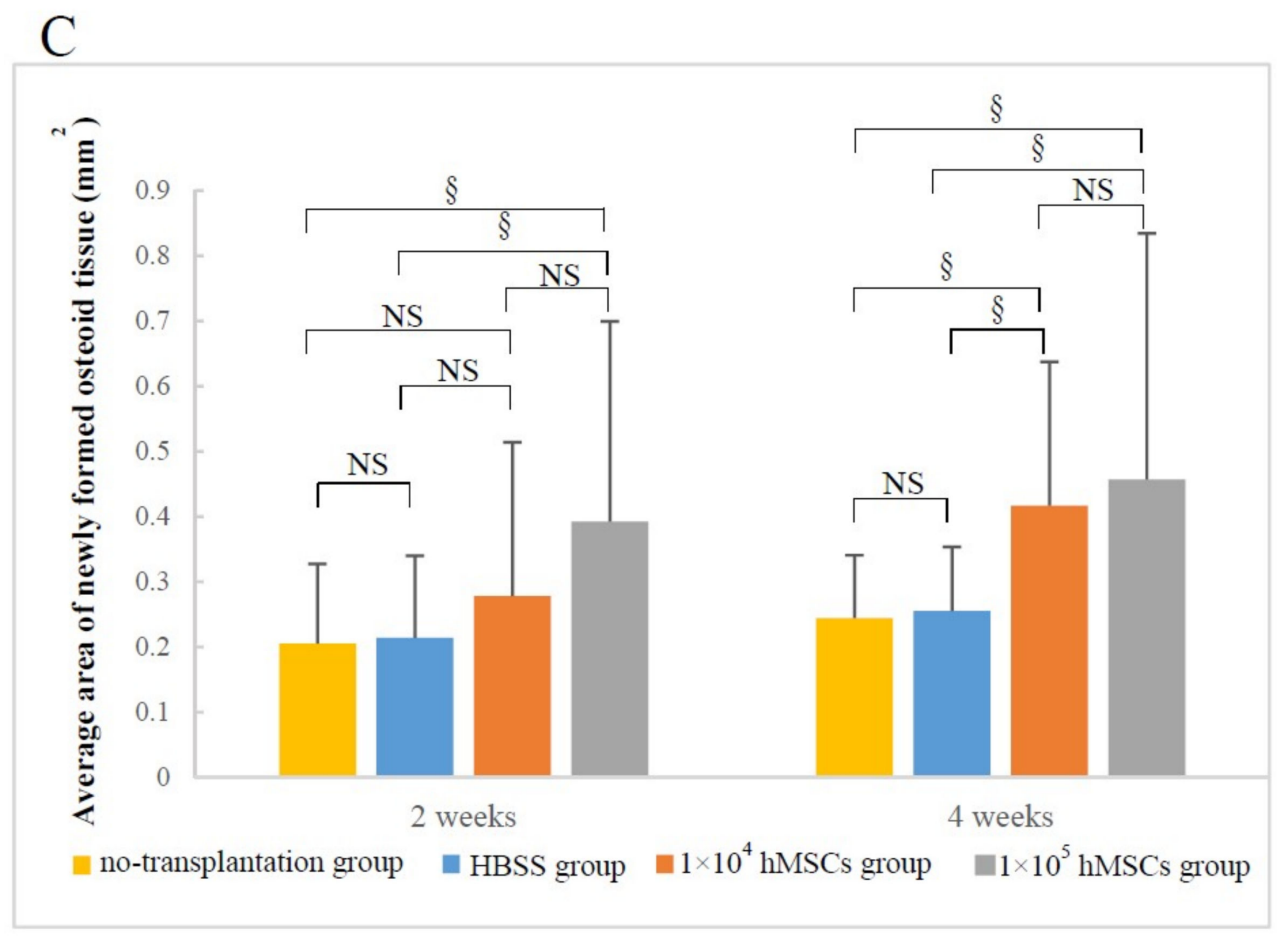
The material–host bone combinations and the amounts of newly formed osteoid tissue based on (**A**) the average fusion rate, (**B**) the average fusion depth, and (**C**) the average area of newly formed osteoid tissue. (**A**,**B**) Analyzed using the Kruskal–Wallis H test; (**C**) Analyzed by one-way analysis of variance and the LSD-*t* test; * *p* < 0.005; ^§^ *p* < 0.05; NS: no significance. The error bars indicate standard deviations.

The average fusion rate and depth of the two hMSCs groups were not only higher than those of the composite only but also increased from two weeks to four weeks after surgery (Figure 5A,B). Furthermore, the average area of newly formed osteoid tissue increased over time in the following order: the no-transplantation group, the HBSS group to the 1 × 10^4^ hMSCs group, and the 1 × 10^5^ hMSCs group (Figure 5C). Although there were no significant differences between the two hMSCs groups in the three indices above at two and four weeks (1 × 10^4^ hMSCs group vs. 1 × 10^5^ hMSCs group: *p* > 0.05), the 1 × 10^5^ hMSCs group showed a slight improvement over the 1 × 10^4^ hMSCs group (Figure 5). Taken together, the results indicated that hMSCs supported the transplantation of 3D-HA/PDLLA.

#### 3.1.3. Difference in Osteogenesis between the Superior and Inferior Sides of the Critical Mandibular Defect

Table 1 and Table 2 show no apparent differences in the average fusion rates and depths of the defection superior side and inferior side at two weeks. However, at four weeks, there were significant differences in both indices between the two sides, revealing improved osteogenesis and extent of fusion on the superior side than the inferior side (average fusion rate: *p* < 0.005; average fusion depth: *p* < 0.006).

### 3.2. VG Staining Results

#### 3.2.1. Description of VG Staining

**Figure 6:**
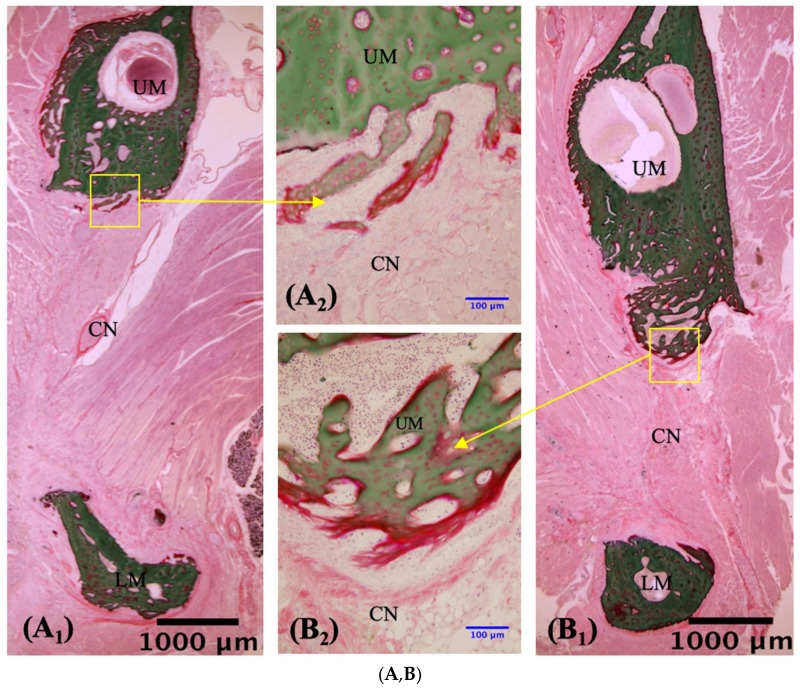

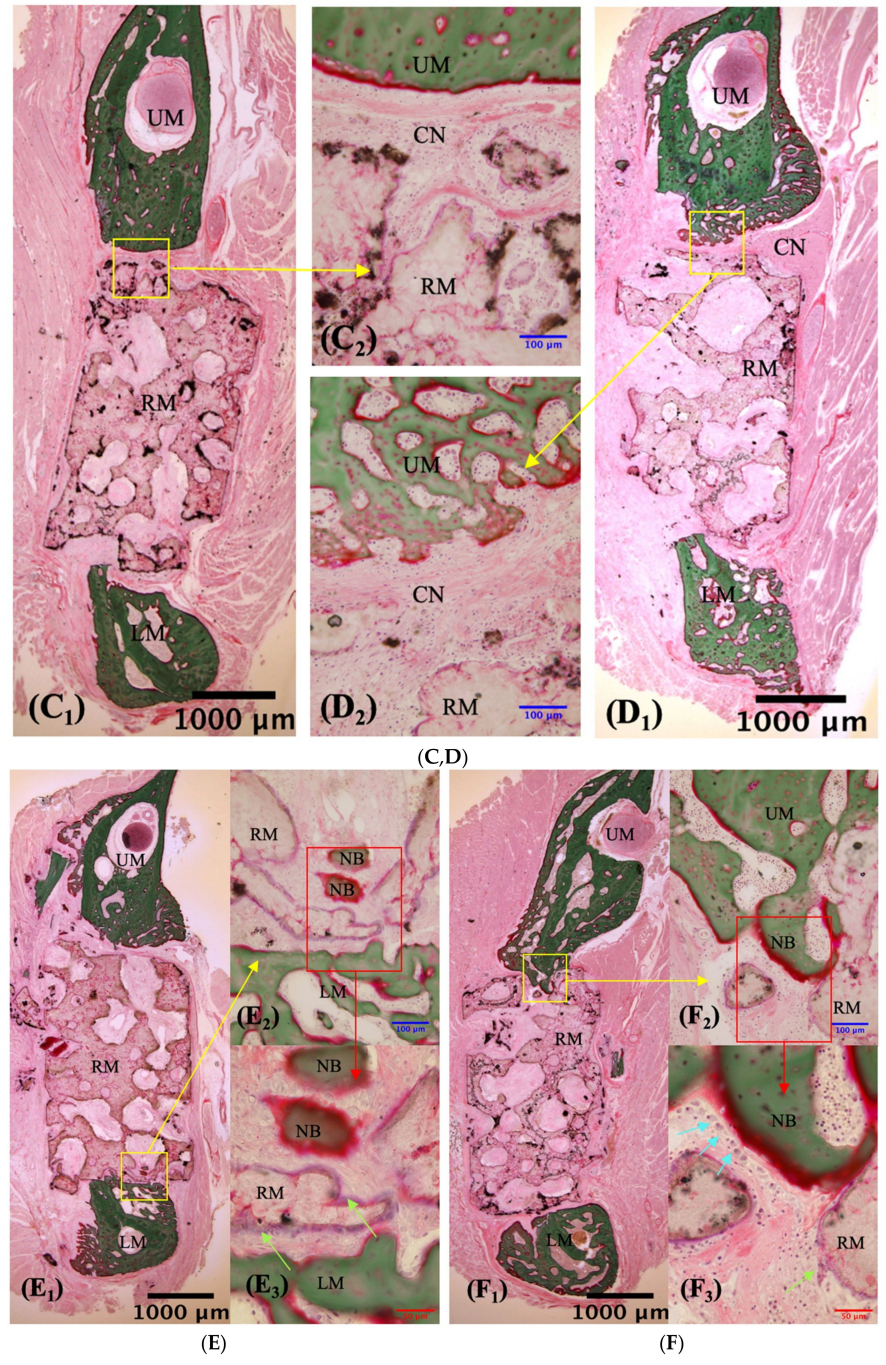

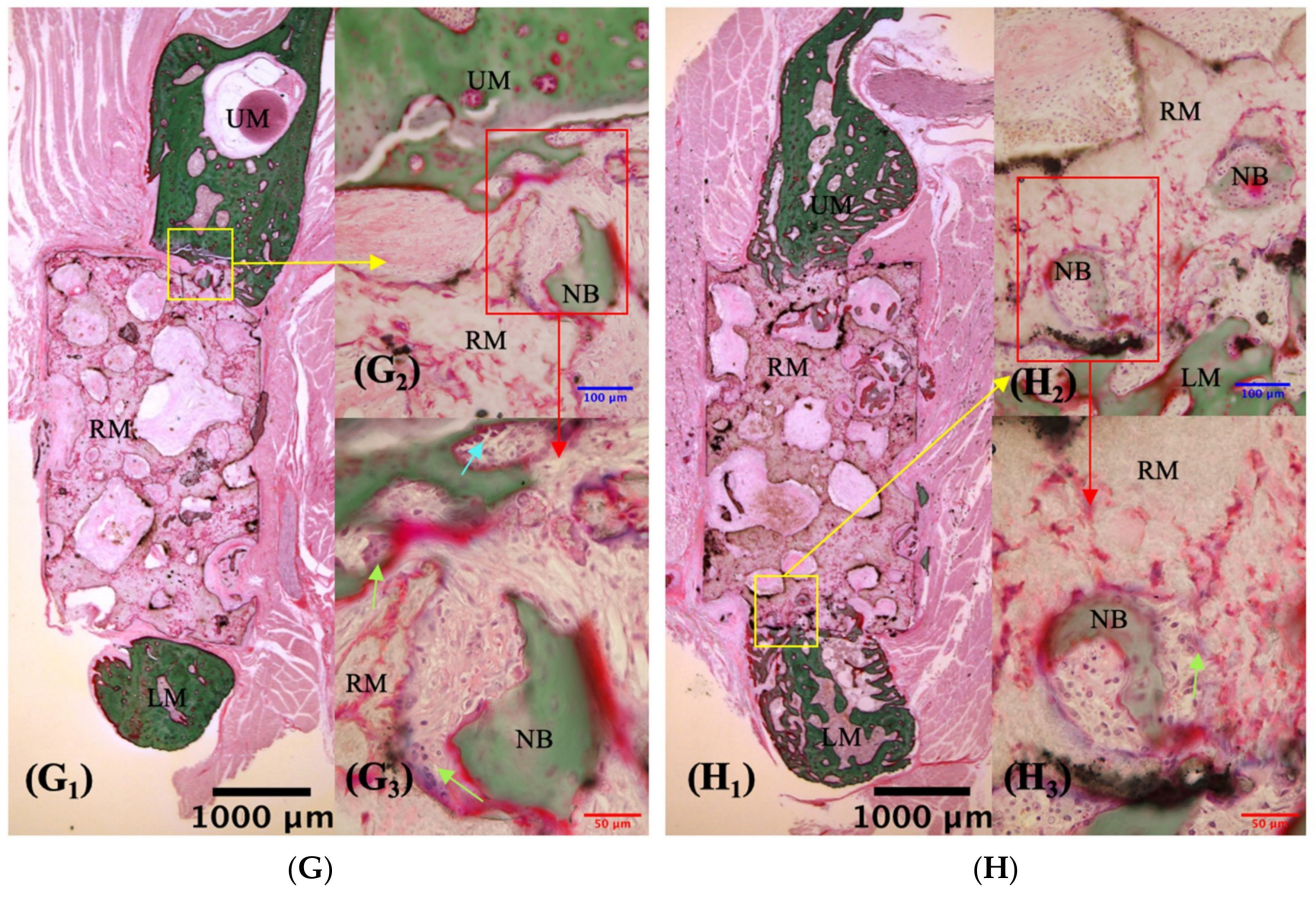
Villanueva Goldner staining of undecalcified sections in the no-transplantation (n = 3), HBSS (n = 3), 1 × 10^4^ hMSCs (n = 3), and 1 × 10^5^ hMSCs (n = 3) groups. ((**A**,**C**,**E**,**G**); n = 3) Slices prepared 2 weeks after creation of the mandibular defect. ((**B**,**D**,**F**,**H**); n = 3) Slices prepared at 4 weeks. Newly formed bone was observed on and in the material shown in panels (**E**–**H**). RM, residual material; UM, upper mandible; LM, lower mandible; NB, newly formed bone; CN, connective tissue. The blue arrows in panels (**F_3_**,**G_3_**) indicate cuboidal osteoblast-like cells that lay in rows adjacent to newly formed bone. The green arrows in panels (**E_3_**,**F_3_**,**G_3_**,**H_3_**) indicate chondrocytes that synthesize the cartilaginous extracellular matrix. (**A_1_**,**B_1_**,**C_1_**,**D_1_**,**E_1_**,**F_1_**,**G_1_**,**H_1_**): ×1.25 magnification. (**A_2_**,**B_2_**,**C_2_**,**D_2_**,**E_2_**,**F_2_**,**G_2_**,**H_2_**): ×20 magnification. (**E_3_**,**F_3_**,**G_3_**,**H_3_**): ×40 magnification. Scale bars: 1000 μm (black), 100 μm (blue), 50 μm (red).

To evaluate the bone regeneration after implantation, VG staining was performed with undecalcified sections of defected mandibles. No or slightly regenerated osteoid tissue was observed around the border region between the composite and mandibular bone in the no-implantation and composite-only implantation controls (Figure 6A–D). In the no-transplantation group, connective and muscle tissue surrounded the margins of the defects at two and four weeks, limiting the growth of osteoid tissue (Figure 6A,B). In the HBSS group, connective tissue connected the material to the margin of the host bone at both two and four weeks. Small amounts of endochondral ossification within the pores were evident at both two and four weeks, but unmineralized bone tissue was observed (Figure 6C,D). Invasion of the implant by newly formed osteoid tissue was clearly observed in the two hMSCs groups (Figure 6E–H) and was also apparent on Micro-CT (Figure 4E–H). In the 1 × 10^4^ hMSCs group, new bone grew into the pores by two weeks (Figure 6E). At four weeks, the junction had closed and new bone had expanded into the 3D-HA/PDLLA composite along with abundant cartilage-like tissue (Figure 6F). In the 1 × 10^5^ hMSCs group at two weeks, new bone had grown into the pores and active osteogenesis was apparent, featuring the presence of a large number of osteoblasts and new calcified bone (Figure 6G). At four weeks, a tight mechanical interlock had formed between the material and host bone. A large amount of new calcified bone had grown into the surrounding pores, along with new blood vessels and clumps of osteoblasts (Figure 6H). At ×40, rows of osteoblasts could be observed surrounding the most active regions of new calcified bone (Figure 6F_3_,G_3_). This bone gradually extended along the inner wall of the material, to replace the cartilage matrix (Figure 6E_3_,F_3_,G_3_,H_3_).

#### 3.2.2. Number of Nucleated Cells in Different Pore Areas

**Figure 7:**
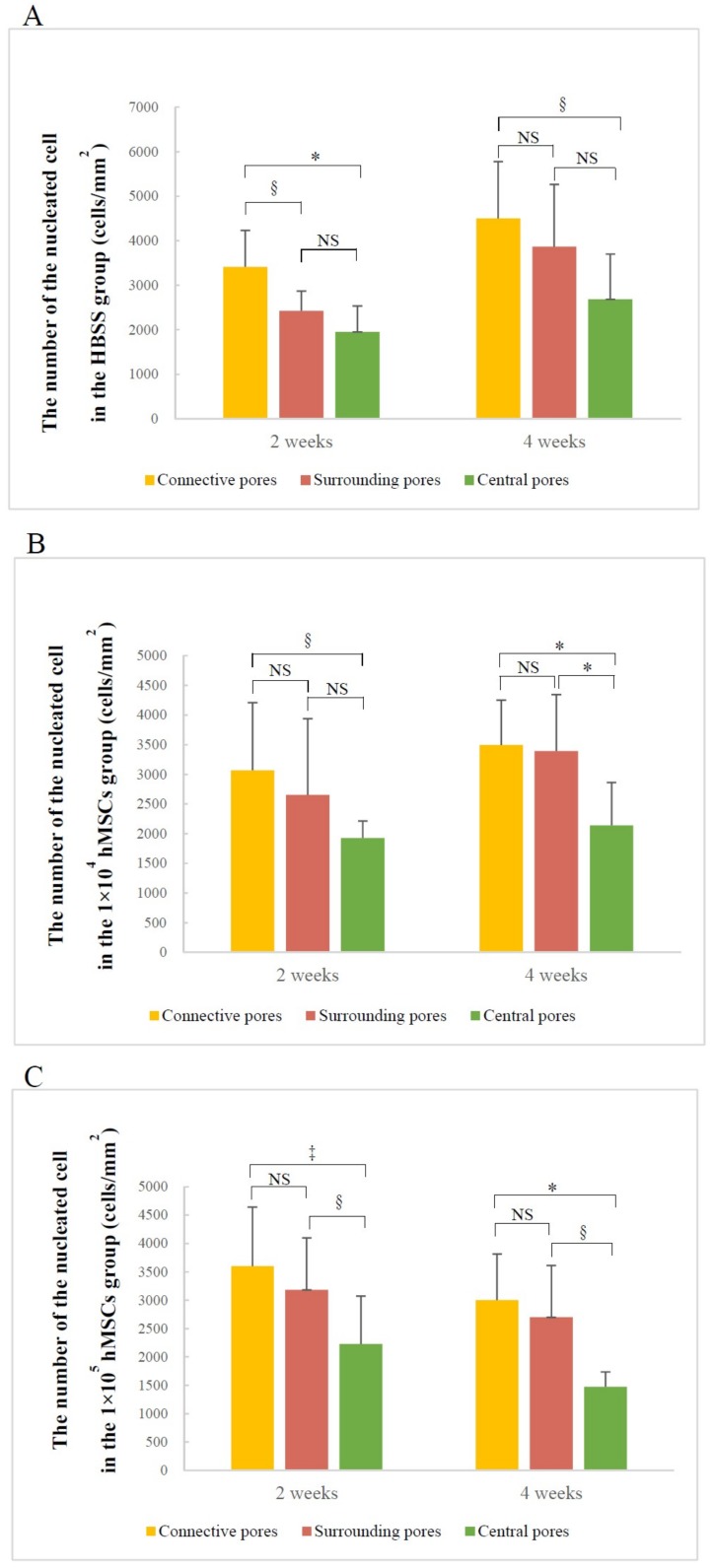

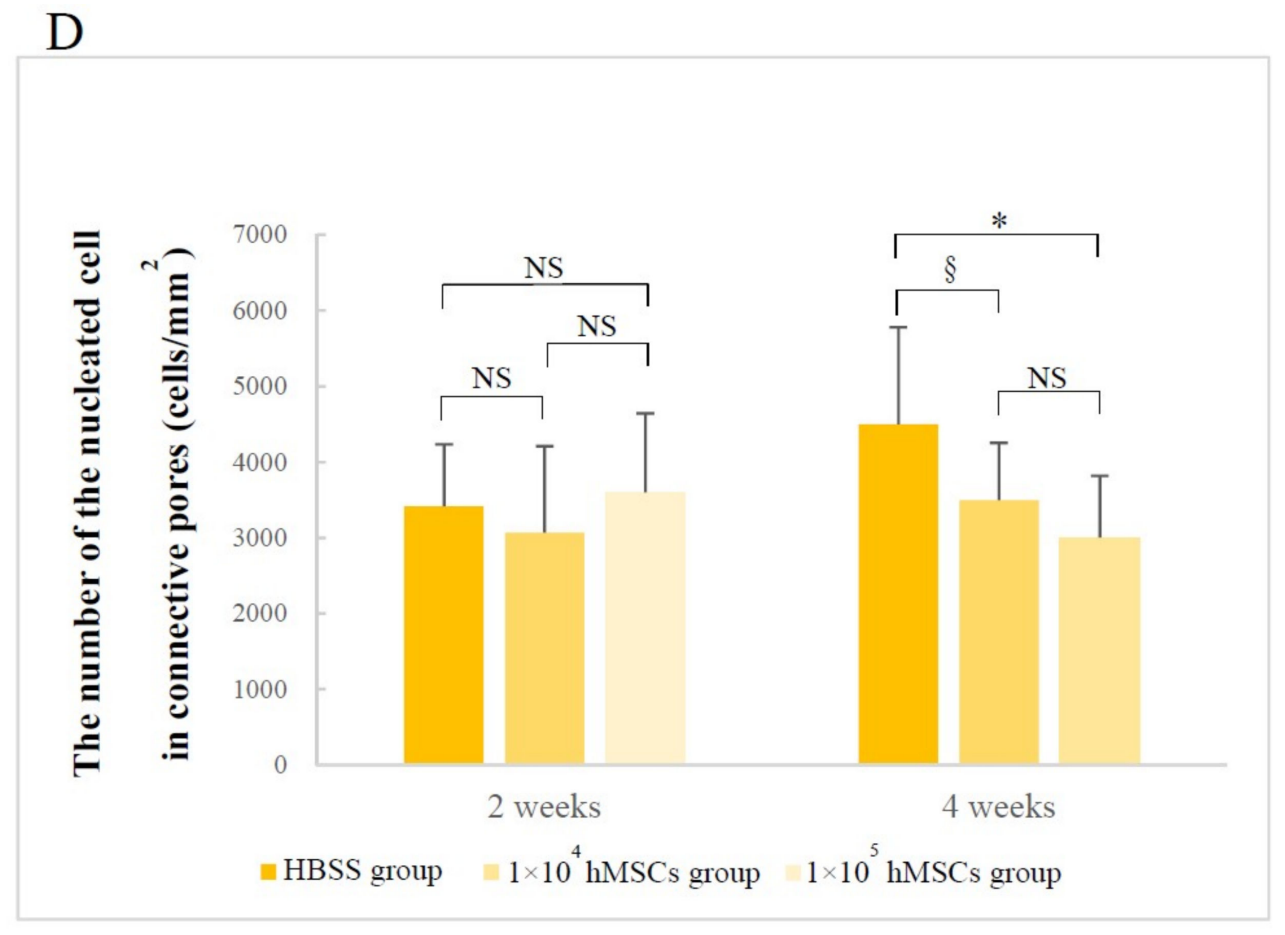
Nucleated cell counts in the pores of different areas estimated using Villanueva Goldner staining. The nucleated cell counts in connective pores, surrounding pores, and central pores in the (**A**) HBSS, (**B**) 1 × 10^4^ hMSCs, (**C**) 1 × 10^5^ hMSCs groups, and (**D**) nucleated cell counts in connective pores only. (**A**,**B**,**C**,**D**) analyzed using one-way analysis of variance and the LSD-*t* test; * *p* < 0.005; ^‡^ *p* < 0.01; ^§^ *p* < 0.05; NS: no significant. Error bars show standard deviations.

The composite pores of the different areas included the same trend of nucleated cell counts. In all groups, the number of nucleated cells decreased gradually from the connective to the central pores, and throughout the experiment. Only the 1 × 10^4^ hMSCs group at four weeks, and 1 × 10^5^ hMSCs group at both two and four weeks exhibited significant differences between the surrounding and central pores (Figure 7A–C). Furthermore, there was no significant difference in connective pore cell counts among the three groups at two weeks; however, at four weeks, there were significantly fewer cells in the two hMSCs groups than the HBSS group (HBSS group vs. 1 × 10^4^ hMSCs group: *p* < 0.05; HBSS group vs. 1 × 10^5^ hMSCs group: *p* < 0.005) (Figure 7D). There was no significant difference between the number of nucleated cells in the surrounding and central pores; therefore, the results are not shown.

## 4. Discussion

Maxillofacial boney defects, particularly critical segmental bone defects, are difficult to restore and reconstruct because the transplantation materials must both withstand strong masticatory pressure and support 3D esthetic requirements. The 3D-HA/PDLLA porous scaffold composites, which best meet the requirements, are being researched for clinical application. Using in vivo experiments, we evaluated the effects of porous 3D-HA/PDLLA-hMSC scaffolds in supporting ectopic osteogenesis. Based on Micro-CT and VG staining, we achieved satisfying material–host bone combinations and quantity of newly formed osteoid tissue, (Figure 5 and Figure 6E–H). These results prove that the 3D-HA/PDLLA scaffold provides a favorable growth environment for hMSCs.

The 3D-HA/PDLLA scaffolds host the growth of hMSCs and mimic bone morphology and function. The pore size and porosity of biomaterial scaffolds play important roles in bone reconstruction. The morphology of the trabecular bone provides a porous environment of 50–90% porosity with a surrounding cortical bone [26]. The average pore density of 70% in the 3D-HA/PDLLA scaffold was similar in structure to the cancellous bone matrix, allowing for the migration and proliferation of mesenchymal cells and osteoblasts, as well as vascularization. The 3D-HA/PDLLA scaffold included different size pores with an average diameter of 40–480 μm. Large pores (diameter > 100 μm) showed substantial bone ingrowth, and the invasion of vascular tissues and nutrients from the surrounding tissues [26,27]. We observed more newly formed osteoid tissue and chondral tissue in the large pores in the two hMSCs groups at two and four weeks (Figure 6E–H). The cell density in medium pores (diameter: 75–100 μm) was higher and filled unmineralized osteoid tissue earlier, because more hMSCs were entrapped to prevent outflow into the surrounding tissues [13]. The large- and medium-diameter pores are optimal for bone conduction (Figure 6E–H). Smaller pores (diameter < 75 μm) penetrated fibrous tissue and produced a matrix formed by osteoblasts, but the vascularization was not obvious. Using SEM, the micromorphology of the 3D-HA/PDLLA was rough (Figure 1B). The rough surface of the pores resulted in a larger surface area, which we thought contributed to higher bone-inducing protein adsorption as well as to ion exchange and bone-like apatite formation by dissolution and reprecipitation [28]. Additionally, the porous structure enhanced interlocking between the implanted biomaterial and the host bone, increasing mechanical stability at this critical interface [29], the 3D-HA/PDLLA-hMSC scaffold resulted in an excellent material–host bone combination. These physical properties allowed the 3D-HA/PDLLA scaffold to provide enough stable space for the differentiation and proliferation of hMSCs. Recently, prototypes testing the mechanical proprieties of scaffolds have been developed, for example, the biomechanical interplay between cancer cells and the surrounding extracellular matrix and the impact on tumor phenotype and behavior [30]. In the future, we will explore the detailed mechanobiology in play.

The chemical composition of a porous biomaterial influences both cell behavior and tissue regeneration [31]. The u-HA is considered a suitable matrix for application of hMSCs, is resorbed into the surrounding natural bone, and showed strong osteoconductivity without causing physical irritation [32]. Given that appropriate concentrations of calcium and phosphate ions may be essential for osteogenesis by osteogenic cells [29], the matrix of 3D-HA/PDLLA provided an appropriate Ca/P ratio to support the growth of new bone. Polylactic acid (PLA) is a semicrystalline polymer that can exist in several distinct forms, such as poly-d-lactide (PDLA) and poly-l-lactide (PLLA), depending on the dextrorotatory (d) and levorotatory (l) configurations; it is also degraded via hydrolysis [33]. PDLLA combines the advantages of PLLA and PDLA. It is the reason that these composites had high enough plasticity to modify the various scaffold shapes and prevented their destruction after implantation; thus, it created a stable environment allowing hMSCs proliferation and differentiation. Previous studies reported that following intramedullary fixation for osteotomy at both four and six weeks, the mRNA and protein expressions of hypoxia-inducible factor 2 alpha (HIF2A) in callus tissue was increased in the PDLLA group compared to the control [34]. This further promotes the expressions of runt-related transcription factor 2 (Runx2) through an interaction between HIF2A and the Runx2-P1 promoter during the process of traumatic bone repair to help hMSCs differentiate into osteoblasts [35]. Overall, reasonable chemical properties determined the higher fusion level and bone formation of the 3D-HA/PDLLA-hMSC scaffolds used in the present study.

hMSCs have recently become a potential cell source for bone tissue repair and regeneration [36]. The other preclinical studies confirmed bone formation using scaffolds as carriers of hMSCs [37,38]. Because the hMSCs possess the osteogenic potential and anti-inflammatory, immunomodulatory, and antiapoptotic properties, hMSCs have become the methods of cell therapy for the treatment of bone and joint diseases [39]. The hMSCs are present in bone marrow stroma and newly formed bone with a bone growth acceleration between 60 and 94% [40]. Direct injection of hMSCs may not aid repair of substantial bone defects or bony nonunion. Therefore, both transplanted hMSCs and an appropriate carrier are essential prerequisites for in vivo bone regeneration [41]. In this study, the effect demonstrated by the 1 × 10^5^ hMSCs group reflected a slight improvement over that demonstrated by the 1 × 10^4^ hMSCs group, indicating that more stem cells resulted in increased bone formation (Figure 5). The hMSCs secrete a broad repertoire of trophic and immunomodulatory factors [42]. It has been reported that hMSCs is mediated by the cooperative effects of cytokines, such as hepatocyte growth factor, transforming growth factor beta-1, insulin-like growth factor-1 [43,44], bone morphogenetic protein-1 [45], and monocyte chemoattractant protein [46], which are involved in cell migration, proliferation, differentiation, osteogenesis, and angiogenesis [43]. In the present study, adding CD90/CD271 double-positive highly purified and superselective hMSCs to the material cubes resulted in more differentiation of osteoblasts and chondrocytes, which may improve the rate of ossification. Therefore, the influence of hMSCs is essential for new bone formation in porous material.

Osteogenesis occurred significantly faster on the superior side of the mandibular defect than on the inferior side (Table 1 and Table 2). This indicated that blood supply and nutrition affected osteogenesis. The mandibular alveolar artery enters the mandible through the mandibular foramen and passes through the mandibular canal. Its branches nourish the mandible body and ascending branch below the foramen. Furthermore, the periosteal vascular network is functional, and blood supply from the alveolar artery and the periosteal vascular network is conducive to the vascularization of tissue-engineered bones [47]. Similar to the mandible in humans, the defect on the superior side of the mandible in rats was closer to the neurovascular bundle, which received a greater blood supply than the inferior side (Figure 3A). This indicated that when using porous material and stem cells to support vascularization, bone formation, and cell proliferation, the supply of adequate nutrients and blood supply is essential. A lack of blood supply causes delayed healing and nonunion at the fracture site [48]. Although constructing scaffold composite is the optimal choice for repairing critical bone defects, the vascularization of newly formed bone depends on the time-consuming ingrowth of peripheral blood vessels. The material-complexed hMSCs and osteoblasts may die before the reestablishment of normal blood circulation [47]. Hence, blood supply should be reestablished to retain the osteogenic activity of the material as soon as possible. We believe that it is necessary to establish an in vitro vascular network that supports bone tissue formation and maxillofacial fracture healing. Although the anatomical nutrition supply cannot be changed, postoperative nutrition supply to fractures is crucial.

We analyzed the nucleated cell count in different areas of pores via VG staining. Overall, the number of cells in the three areas decreased from connective pores to central pores in all specimens (Figure 7A–C). The formation of osteogenically relevant cells and new bone was initiated in connective and surrounding areas and gradually shifted to the central area. This indicates that regardless of the specific group, the direction of cell migration was consistent with that of osteogenesis in the 3D-HA/PDLLA scaffold. This phenomenon may be influenced by osteoinductive proteins, which promote the differentiation of hMSCs, migrating from surrounding tissue into osteoblasts actively, which are capable of bone regeneration [49]. Scaffolds serve primarily as osteoconductivity because the newly formed bone is deposited by creeping substitution from adjacent host bone [50]. If the hMSCs were cultured in vitro in the 3D-HA/PDLLA scaffold in advance, the number of hMSCs in the material might increase to achieve a better osteogenic effect. However, it is also possible that the lack of nutritional support in the central area may trigger apoptosis prior to cell proliferation. Meanwhile, the HBSS group exhibited more nucleated cells in connective pores than either of the two hMSCs groups at four weeks (Figure 7D), which occurred because a given amount of bone matrix and calcification of bone were generated in the two hMSCs groups. The proliferation rates of nucleated cells in the two hMSCs groups peaked at two weeks and then began to decrease, because the decrease provided more space for new bone growth. However, this phenomenon did not occur in surrounding and central pores, because the new bone had not filled enough space at four weeks. Therefore, the longer periods are required to achieve satisfying results. Overall, the nucleated cell count showed that the 3D-HA/PDLLA-hMSC scaffold exhibited better osteoconductivity.

In this in vivo study, injection of an adequate number of hMSCs accelerated the proliferation and differentiation of osteoblasts and the restoration of defects within 3D-HA/PDLLA scaffolds. This indicated that a combination of 3D-HA/PDLLA and hMSCs was optimal for the reliable, active promotion of maxillofacial bone reconstruction. The results can be used to support future clinical applications of 3D-HA/PDLLA-hMSC scaffolds for maxillofacial boney defect reconstruction.

## 5. Conclusions

The 3D-HA/PDLLA-hMSC scaffold in a mandibular defect improved the bone formation compared to 3D-HA/PDLLA scaffold filling alone. The growth of osteogenesis-relevant cells and new bone gradually progressed from connective pores to central pores, demonstrating that the 3D-HA/PDLLA-hMSC scaffold exhibited a better osteoconductivity. Given an adequate blood supply, the 3D-HA/PDLLA-hMSC scaffold effectively aids bone regeneration for boney defect reconstruction in maxillofacial surgery.

## Figures and Tables

**Table 1:** Average fusion rates of the superior and inferior sides of the critical mandibular defects.

Time	Superior Side Mean ± S.D. (%)	Inferior Side Mean ± S.D. (%)	Z Value	*p*-Value
2 weeks	23.61 ± 23.65	25.04 ± 24.95	0.370	0.711
4 weeks	35.91 ± 26.61	15.54 ± 19.16	3.611	0.005

The Wilcoxon signed-rank test was used.

**Table 2:** Average fusion depths of the superior and inferior sides of the critical mandibular defects.

Time	Superior Side Mean ± S.D. (mm)	Inferior Side Mean ± S.D. (mm)	Z Value	*p*-Value
2 weeks	0.256 ± 0.248	0.191 ± 0.225	1.264	0.206
4 weeks	0.458 ± 0.449	0.265 ± 0.245	2.749	0.006

The Wilcoxon signed-rank test was used.

## Abbreviations

3D	three-dimensional;
3D-HA/PDLLA	three-dimensional porous uncalcined and unsintered hydroxyapatite/poly-d/l-lactide;
MSCs	mesenchymal stem cells;
hMSCs	human mesenchymal stem cells;
β-TCP	β-tricalcium phosphate
BMA	bone marrow aspirate
CD90	Thy-1, cluster of differentiation 90
CD271	low-affinity nerve growth factor receptor
MECs	moderately expanding mesenchymal stem cell clones
u-HA	uncalcined and unsintered hydroxyapatite
PDLLA	poly-d/l-lactide
Mv	viscosity-average molecular weight
d/l	dextrorotatory-lactide acid/levorotatory-lactide acid
Ca/P	calcium/phosphorus
HBS	Hank’s balanced salt solution
SD	Sprague Dawley
Micro-CT	microcomputed tomography
VG staining	Villanueva Goldner staining
PLA	polylactic acid
PLLA	poly-l-lactide
PDLA	poly-d-lactide
d	dextrorotatory
l	levorotatory
HIF2A	hypoxia-inducible factor 2 alpha
Runx2	runt-related transcription factor 2
